# Targeting and Imaging of Cancer Cells via Monosaccharide-Imprinted Fluorescent Nanoparticles

**DOI:** 10.1038/srep22757

**Published:** 2016-03-07

**Authors:** Shuangshou Wang, Danyang Yin, Wenjing Wang, Xiaojing Shen, Jun-Jie Zhu, Hong-Yuan Chen, Zhen Liu

**Affiliations:** 1 State Key Laboratory of Analytical Chemistry for Life Science, School of Chemistry and Chemical Engineering, Nanjing University, Nanjing 210023, China; 2 Collaborative Innovation Center of Chemistry for Life Sciences, School of Chemistry and Chemical Engineering, Nanjing University, Nanjing 210023, China

## Abstract

The recognition of cancer cells is a key for cancer diagnosis and therapy, but the specificity highly relies on the use of biorecognition molecules particularly antibodies. Because biorecognition molecules suffer from some apparent disadvantages, such as hard to prepare and poor storage stability, novel alternatives that can overcome these disadvantages are highly important. Here we present monosaccharide-imprinted fluorescent nanoparticles (NPs) for targeting and imaging of cancer cells. The molecularly imprinted polymer (MIP) probe was fluorescein isothiocyanate (FITC) doped silica NPs with a shell imprinted with sialic acid, fucose or mannose as the template. The monosaccharide-imprinted NPs exhibited high specificity toward the target monosaccharides. As the template monosaccharides used are over-expressed on cancer cells, these monosaccharide-imprinted NPs allowed for specific targeting cancer cells over normal cells. Fluorescence imaging of human hepatoma carcinoma cells (HepG-2) over normal hepatic cells (L-02) and mammary cancer cells (MCF-7) over normal mammary epithelial cells (MCF-10A) by these NPs was demonstrated. As the imprinting approach employed herein is generally applicable and highly efficient, monosaccharide-imprinted NPs can be promising probes for targeting cancer cells.

Specific recognition of cancer cells is a key for cancer diagnosis and therapy. Antibodies have been the workhorses for the recognition of cancer cells^1^,^2^,^3^. In addition, aptamers^4^,^5^, peptides^6^,^7^, and lectins^8^,^9^ have already emerged as important alternatives. However, all these biomolecules suffer from some disadvantages. For instance, antibodies and lectins are hard to prepare, poor in storage stability and susceptible to protease degradation, while aptamers and peptides are generally associated with relatively poor specificity and risk of degradation. Therefore, novel alternatives that can overcome these disadvantages are highly important.

Molecularly imprinted polymers (MIPs)^10^,^11^,^12^,^13^,^14^ are chemically synthetic receptors with predesigned binding specificity and affinity toward to target molecules. The molecular imprinting process usually involves initiating the polymerization of functional monomers and cross-linker in the presence of a template (the target) that is extracted afterwards, thereby leaving complementary cavities in the polymer matrix. As compared with biomolecules such as antibodies, MIPs are easy to prepare, cost-efficient and more stable. MIPs have found important applications in many areas such as chemical sensing^15^, separation^16^, catalysis^17^, and disease diagnostics^18^,^19^,^20^. To recognize cells, a conventional imprinting strategy is to use target cells directly as template^21^,^22^,^23^. Although some promising applications such as blood typing^21^ and programmed cell adhesion/growth^22^ have been demonstrated, recognizing cancer cells by cell-imprinted MIPs is challenging due to change in the surface nature and shape of cancer cells.

Altered glycosylation is a universal feature of cancer cells, and aberrant expression of certain glycan structures are well-known markers for recognizing cancer cells. For instance, sialylation^24^,^25^ and fucosylation^26^,^27^ are over-expressed on the cell surface of most cancers, while mannosylation is over-expressed on the cell surface of certain cancers such as liver cancer^28^,^29^. Recently, a new imprinting strategy has been proposed for the preparation of MIPs for cell recognition, which used monosaccharides expressed on cell surface as the templates. Haupt and co-workers^30^ first demonstrated the application of fluorescently labeled glucuronic acid-imprinted nanoparticles (NPs) for cell and tissue imaging. Sellergren and co-workers^31^ further reported sialic acid (SA)-imprinted fluorescent NPs for selective labeling of cell surface glycans. Very recently, we reported SA-imprinted NPs for surface enhanced Raman scattering (SERS) imaging of cancer cells and tissues over normal cells and tissues^32^. In the two SA-imprinted MIPs, boronic acids, which can reversibly interact with cis-diol-containing molecules such as sugars^33^,^34^, were used as a functionality. Although some boronic acids such as phenylboronic acid were reported to be able to differentiate sialic acid (SA) and other monosaccharides^35^,^36^ and thereby have been used to target cancer cells^37^,^38^, our experimental evidence revealed that such the recognition is not robust and MIP is much superior to boronic acid-functionalized materials^32^. The monosaccharide imprinting strategy opened a new avenue for the recognition of cells. However, further in-depth exploration is much needed. Particularly, it is critical to verify whether such a strategy is widely applicable for the recognition of cancer cells over normal cells and for more monosaccharide templates. If the answers are yes, then a generally applicable and facile approach for monosaccharide imprinting is highly desirable.

In this study, we confirmed that monosaccharide-imprinted MIPs can be used as a general toolbox for the specific recognition of cancer cells. We also confirmed that the boronate affinity oriented surface imprinting approach developed recently allows for facile and efficient preparation of monosaccharide-imprinted MIPs. Fluorescent monosaccharide-imprinted NPs were prepared and application in targeting and fluorescence imaging of cancer cells was demonstrated. The principle of recognition and imaging of cancer cells is illustrated in Fig. 1 and the synthesis route of monosaccharide-imprinted NPs is schematically shown in Fig. 2. SA, fucose (Fuc) and mannose (Man) were selected as the templates individually. Fluorescein isothiocyanate (FITC) doped silica NPs were first synthesized as a fluorescent core and further functionalized with a monosaccharide-imprinted silica layer by the boronate affinity oriented surface molecular imprinting^32^,^39^,^40^,^41^. The obtained monosaccharide-imprinted NPs were verified to be able to specifically recognize target monosaccharide and to differentiate cancer cells from normal cells. Fluorescence imaging of human hepatoma carcinoma cells (HepG-2) over normal hepatic cells (L-02) and mammary cancer cells (MCF-7) over normal mammary epithelial cells (MCF-10A) by these NPs was demonstrated.

## Results and Discussion

### Characterization of Monosaccharide-imprinted NPs

As compared with fluorescent molecules, fluorescent NPs exhibit several advantages, such as more stable fluorescence, better signal-to-noise ratio, higher sensitivity and better biocompatibility^42^,^43^. Due to its strong fluorescence and ease in grafting, FITC was selected as a fluorescence reporter. Fluorescent silica NPs were prepared through polycondensation of TEOS in the presence of FITC-modified APTES. Fluorescent NPs prepared by this means have several advantages, including stable fluorescence, facile post-modification and good water dispersity. The prepared FITC-doped SiO_2_ NPs showed an absorbance band at 495 nm (Fig. S1), indicating that FITC has been successfully doped into the SiO_2_ NPs. The FITC-doped silica NPs exhibited almost the same fluorescence spectrum as free FITC molecules did (Fig. S2). Leakage test was carried out and the fluorescence intensity (emission wavelength, 520 nm; excitation wavelength, 495 nm) at different times is shown in Fig. S3, which suggested that the fluorescent NPs had good mechanical stability. Photostability test (Fig. S4) revealed that FITC-doped silica NPs possessed much better photostability than free FITC molecules. After continuous excitation at 495 nm for 1 hour, the percentage of photobleaching for FITC-doped silica NPs and FITC molecules was found to be 2.0 and 34.2%, respectively. Furthermore, the fluorescent NPs showed strong fluorescence around physiological pH (Fig. S5). These features ensured high-quality cell imaging under real conditions.

Template immobilization was found to be critical for the formation of monosaccharide-imprinted silica layer. When the template molecules were added into the monomer solution to prepare SA-imprinted NPs via a one-pot process, no SA-imprinted SiO_2_ thin-layer was formed. A reason for this might be that the presence of a relatively large amount of SA in the prepolymer solution significantly reduced the pH of the prepolymer solution and consequently dramatically slowed down the polymerization speed. The boronate affinity of the boronic acid-functionalized silica NPs was evaluated. As shown in Fig. S7, the boronic acid-functionalized silica NPs exhibited apparent boronate affinity toward glucose, suggesting successful boronic acid-functionalization of the fluorescent SiO_2_ NPs. The thickness of the imprinting coating is critical in boronate affinity oriented surface molecular imprinting^32^,^39^,^40^,^41^. Favorably, the thickness generated by the imprinting procedure employed herein is precisely adjustable through changing imprinting time^41^. Thus, the imprinting time for the three monosaccharide templates was optimized in terms of imprinting factor. The optimal imprinting time was found to be 20, 15 and 15 min for SA, Fuc and Man, respectively, which gave an imprinting factor of 8.4, 6.7 and 6.9, respectively (Fig. S8). Such imprinting factor is apparently higher than that for glucuronic acid-imprinted MIP (3.2)^30^. According to the thickness-imprinting time relationship established previously (*y* = 0.04 *x* + 0.51, where *y* is in nm and *x* is in min)^41^, the thickness of the imprinting layer under the optimal imprinting time was estimated to be 1.31, 1.11 and 1.11 nm for SA-, Fuc- and Man-imprinted NPs, respectively, which occupied 81, 88 and 85% of the sum of the estimated molecular length of the monosaccharide template and formylphenylboronic acid (1.02, 0.66, 0.70 and 0.60 nm for SA, Fuc, Man and formylphenylboronic acid, respectively). The high imprinting factors ensured the specificity of the prepared monosaccharide-imprinted silica NPs.

Transmission electron microscopy (TEM) characterization showed that both SA-imprinted and non-imprinted NPs possessed uniform morphology and good water dispersity (Fig. 3A and Fig. S9). Dynamic light scattering (DLS) characterization indicated that the particle sizes of SA-imprinted and non-imprinted NPs were approximately 50 nm and the size distribution was homogeneous (Fig. 3A and Fig. S10). Figure 3B shows the fluorescence spectrum of SA-imprinted NPs. Figure S11 shows the binding isotherms of SA-imprinted and non-imprinted NPs (because Fuc and Man have no UV absorbance, binding isotherms for Fuc and Man are impossible by similar experiments). SA-imprinted NPs exhibited much higher binding strength toward the template as compared with non-imprinted NPs. The *K*_d_ value was estimated to be 2.0 × 10^−4 ^M or 0.071 mg/mL, which is lowered by 2 orders of magnitude as compared with the binding between SA and phenylboronic acid in free solution^35^,^36^. Although there is only one functionality within the MIPs prepared in this study, the binding constant is comparable to that in 98% water for the SA-imprinted MIP prepared by a hybrid approach with trinary functionalities combining boronate affinity binding, electrostatic interaction and hydrogen bonding^31^.

The specificity of the monosaccharide-imprinted NPs toward target monosaccharides was investigated. As shown in Fig. 4, the monosaccharide-imprinted FITC-doped silica NPs exhibited excellent specificity toward the target monosaccharides, with cross-reactivity toward non-target monosaccharides less than 7.1, 26.2 and 22.2% for SA-, Man- and Fuc-imprinted NPs, respectively. Large cross-reactivity of Man- and Fuc-imprinted NPs was due to the structural similarity of Man and Fuc. Such cross-reactivity is much lower than that for glucuronic acid-imprinted MIP (as high as 58%)^30^.

The imprinting efficiency was estimated to be 43.4% for SA, which is outstanding for small molecular imprinting. Dynamic assay (Fig. S12) indicated that the binding equilibrium could be reached within 30 min, which is favorable for the integrity of cells during incubation.

### Specific Targeting of Cancer Cell by SA-imprinted NPs

HepG-2 and L-02 cells were first chosen as model cells. Flow cytometry (FCM) was first used to investigate if the SA-imprinted NPs can selectively recognize cancer cells. After stained with non-imprinted NPs, both HepG-2 and L-02 cells exhibited weak fluorescence; in comparison, after staining with SA-imprinted NPs, both cell lines exhibited obvious fluorescence but HepG-2 cells exhibited stronger fluorescence than L-02 cells (Fig. 5). After staining with SA-imprinted NPs, mixtures containing HepG-2 and L-02 cells at different ratios displayed two peaks: peak 1 with weak fluorescence while peak 2 with strong fluorescence. The peak area ratios of the two peaks were roughly proportional to the cell number ratio and thereby peaks 1 and 2 are assigned to L-02 and HepG-2, respectively. Clearly, cancer and normal cells were differentiated by the SA-imprinted NPs due to the differential binding.

### Cell Imaging via Monosaccharide-imprinted NPs

SA, Fuc and Man-imprinted FITC-doped silica NPs were then used as fluorescent probes for fluorescence imaging of cancerous cells (HepG-2 and MCF-7), with their noncancerous counterparts (L-02 and MCF-7) as controls. As shown in Fig. 6, after staining with the three monosaccharide-imprinted NPs, HepG-2 cells exhibited strong fluorescence and their shapes were apparently fluorescently displayed, while L-02 cells just showed few weak discrete fluorescent dots. In contrast, after staining with non-imprinted FITC-doped NPs, both HepG-2 and L-02 cells exhibited almost no fluorescence (Fig S13–S15). After staining with these MIP NPs, MCF-7 cells exhibited weak fluorescence and their profiles were fluorescently visible, while MCF-10A cells showed no fluorescence. In contrast, after staining with non-imprinted FITC-doped NPs, both MCF-7 and MCF-10A cells exhibited no fluorescence at all (Figs S16–S18). These results clearly indicate that monosaccharide-imprinted NPs permitted selective imaging of cancer cells from normal cells. Besides, the fluorescence intensity in the images reflected the different monosaccharide expression on different cancer cell types. HepG-2 cells exhibited comparable expression level of SA, Fuc and Man while MCF-7 cells exhibited much lower expression level of Fuc and Man than that of SA.

In the imprinting approach used, free monosaccharides were used as templates. The resulting MIPs can preferentially bind with target monosaccharides located on the terminal and they can poorly bind with internal monosaccharides due to stereo-hindrance effect. In biological systems, sialic acid is typically located on the terminal of glycan conjugates (such as glycoproteins and glycolipids), fucose is predominantly located on the terminal while mannose may locate on the terminal or the internal of glycan conjugates. When mannose is over-expressed on cancer cell surface, mannose is existing in two glycan forms, oligomannose and hybrid N-glycans^28^,^29^,^44^. In both cases, although there are internal mannose moieties in the over-expressed glycans, there are also terminal mannose moieties. Therefore, Man-imprinted NPs can target cancer cells through binding with over-expressed terminal mannose.

### Comparison with Fluorescein-labeled Lectins

Fluorescein-labeled lectins that can target SA, fucose and mannose respectively were used as cell staining reagents for comparison. Fluorescence imaging of the cancer cells HepG-2 and MCF-7 against their normal counterparts L-02 and MCF-10A cells was performed. As shown in Fig. S19, the cancer cell-targeting capability of these lectins are similar to that of monosaccharide-imprinted NPs. Also, the relative expression of the monosaccharides on these cancer cells reflected by the fluorescence intensity are also in good agreement with that by imprinted NPs. These results confirmed that monosaccharide-imprinted NPs could provide comparable cancer cell-targeting capability. However, compared with lectins, the monosaccharide-imprinted NPs prepared in this study are advantageous in several aspects. First, these monosaccharide-imprinted NPs are easy to prepare and of low cost. Second, the storage stability and photo-stability of the monosaccharide-imprinted NPs are much better. Third and more encouragingly, the monosaccharide-imprinted NPs could provide better specificity. For instance, the lectin used for the targeting of mannose, *Lens culinaris* agglutinin, can bind not only mannose but also glucose^45^,^46^. As a comparison, the Man-imprinted NPs exhibited very limited cross-reactivity toward glucose (Fig. 4B).

### Specificity Confirmation through Glycosidase Treatment

The specificity of the monosaccharide-imprinted NPs towards the target monosaccharides can be further confirmed through checking the MIP-based cell imaging after cutting off the target monosaccharides from the cell surface by corresponding glycosidases, including sialidase, fucosidase and mannosidase. HepG-2 and MCF-7 cells were used as model cancer cells for this purpose, while their normal counterparts, L-02 and MCF-10A cells, were employed as controls. As shown in Fig. S20, after the target monosaccharides on the cell surface were removed, all the monosaccharide-imprinted NPs failed to stain the cells, no matter they were cancer cells or normal cells. These results indicate that the cell staining capability of the molecularly-imprinted fluorescent NPs was due to their specific binding with the target monosaccharides.

### Recognition of Cancer Cells by Boronic Acid-functionalized NPs

Possibility of using boronic acid-functionalized materials for the recognition of cancer cells was investigated. It was observed that boronic acid-functionalized FITC-doped silica NPs failed to recognize cancer cells from normal cells in both fluorescence microscopy and FCM (Figs S21 and S22). This can be explained by the relatively close binding strengths of boronic acids toward SA and other monosaccharides at physiological pH^32^,^35^.

### Tolerance of Monosaccharide-imprinted NPs to Competing Sugars

As biological systems and samples usually contain free sugars, it is necessary to investigate the tolerance capability of monosaccharide-imprinted NPs to competing sugars. When SA-imprinted NPs were used as a probe meanwhile SA was added to the sample, both HepG-2 and L-02 cells exhibited apparently reduced fluorescence (Fig. 7 and Fig. S23), suggesting that the recognition toward cancer cells was due to the specificity of the MIPs toward the target monosaccharides. When stained with SA-imprinted NPs in the presence of Fuc and Man, both HepG-2 and L-02 cells still displayed strong fluorescence (Fig. 7 and Fig. S23). In comparison, when the boronic acid-functionalized NPs were used as a probe meanwhile a monosaccharide (SA, Fuc or Man) of comparable concentration was added to the cell samples, both HepG-2 and L-02 cells exhibited almost no fluorescence (Fig. S24). Clearly, the monosaccharide-imprinted NPs showed better resistance to the interference of sugars than the boronic acid-functionalized NPs.

## Conclusion

Fluorescent MIPs with three monosaccharides as the template individually have been prepared according to the boronate affinity oriented surface imprinting approach. These monosaccharide-imprinted MIPs exhibited desirable binding properties and could specifically target cancer cells over normal cells. Application in fluorescence imaging of cancer cells was well demonstrated. The imprinting approach was widely applicable to monosaccharides because of the use of boronic acid functionality for one hand. On the other hand, the sol-gel based imprinting approach is very suitable for the imprinting of monosaccharides due to their abundant hydroxyl groups. Meanwhile, Due to the fine-controllability of TEOS condensation, the imprinting approach was facile, requiring only fine-tuning the imprinting time for different monosaccharides. Moreover, it was highly efficient, providing high specificity, high affinity and high imprinting efficiency. Therefore, we foresee monosaccharide-imprinted MIPs can be promising probes in many important applications. For example, monosaccharide-imprinted fluorescent NPs can be applied to tissue imaging for pathological investigation. Besides, monosaccharide-imprinted MIPs can be extended to other response mechanisms. For example, monosaccharide-imprinted plasmonic NPs can be useful probes for targeted photothermal therapy.

## Methods

### Preparation of Monosaccharide-imprinted FITC-doped Silica Nanoparticles

**Figure 2:**
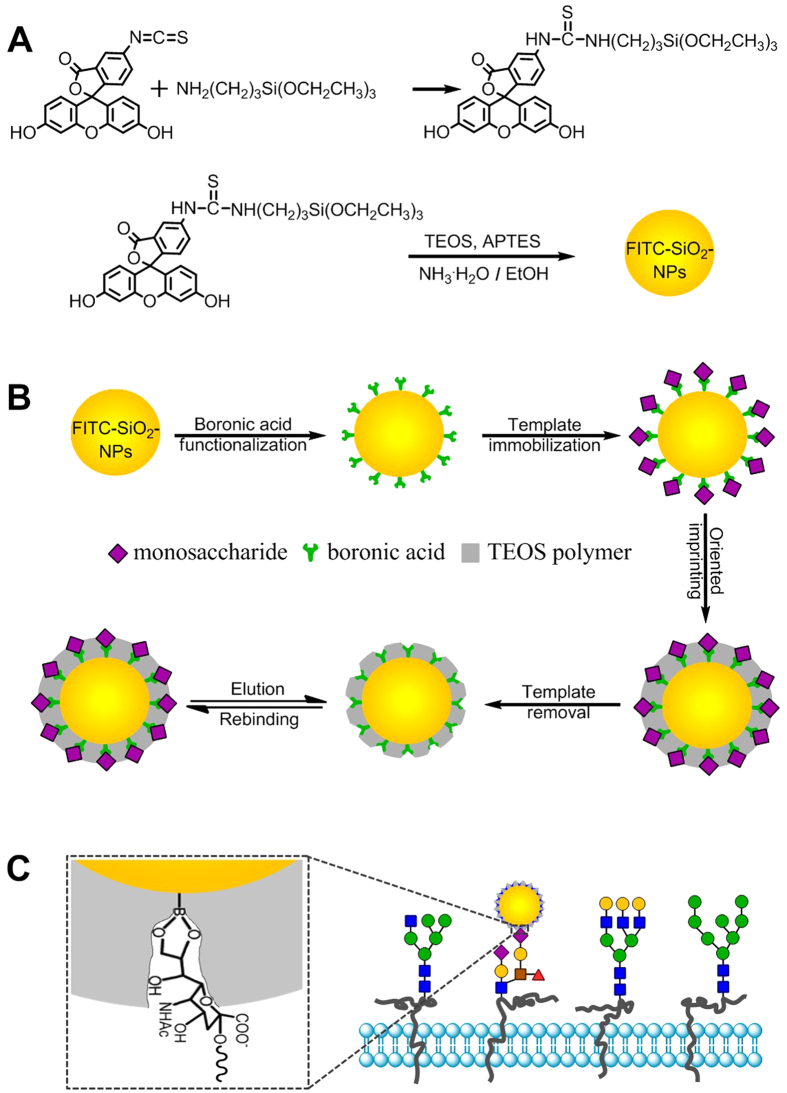
Schematic of the synthesis route of FITC-doped silica NPs (**A**), monosaccharide-imprinted FITC-doped silica NPs (**B**) and the scheme to illustrate the interaction between monosaccharide-imprinted NPs and cells (**C**), illustrated with SA as an example.

**Figure 3:**
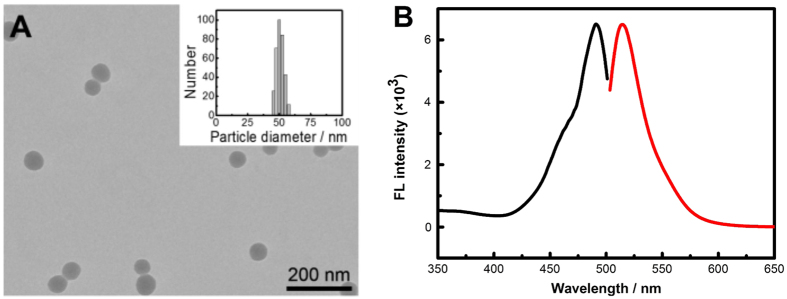
TEM characterization (**A**) and fluorescence spectra (**B**) of SA-imprinted NPs (black trace: excitation; red trace: emission). Insert in A is the particle size characterization of SA-imprinted NPs by dynamic light scattering.

**Figure 4:**
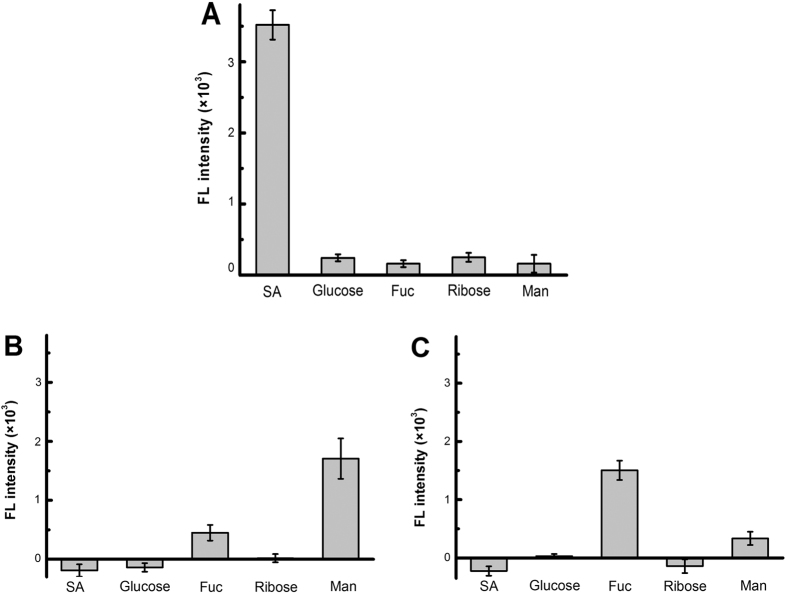
Selectivity of SA- (**A**), Man- (**B**) and Fuc-imprinted NPs (**C**) toward monosaccharides estimated through boronate affinity sandwich assay.

**Figure 5:**
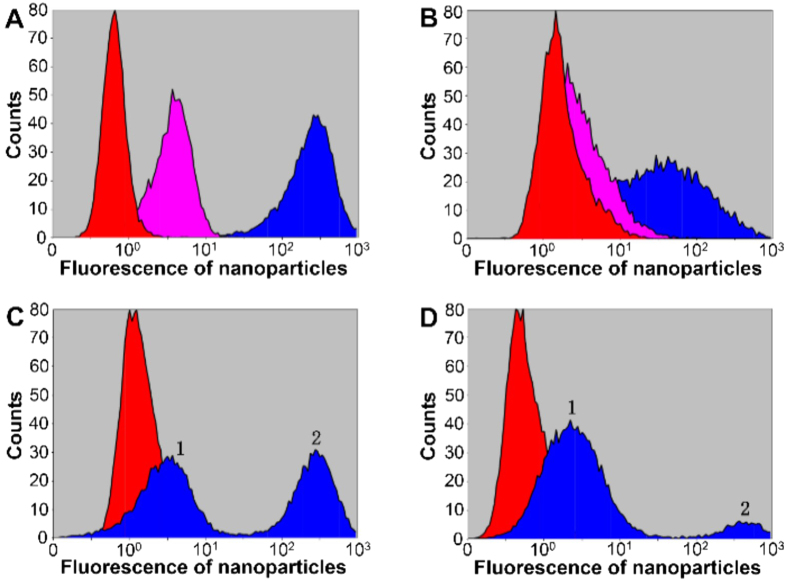
FCM characterization of HepG-2 cells (**A**), L-02 cells (**B**) and a mixture containing HepG-2 and L-02 cells (**C**,**D**) after staining with different materials. Red: without staining (controls). Pink: staining with non-imprinted NPs; blue: staining with SA-imprinted NPs. The cell number ratio of HepG-2 to L-02 for C and D was 1:1 and 1:5, respectively. Peaks 1 and 2 are assigned to L-02 and HepG-2, respectively, according to the peak area ratio and the cell number ratio.

**Figure 6:**
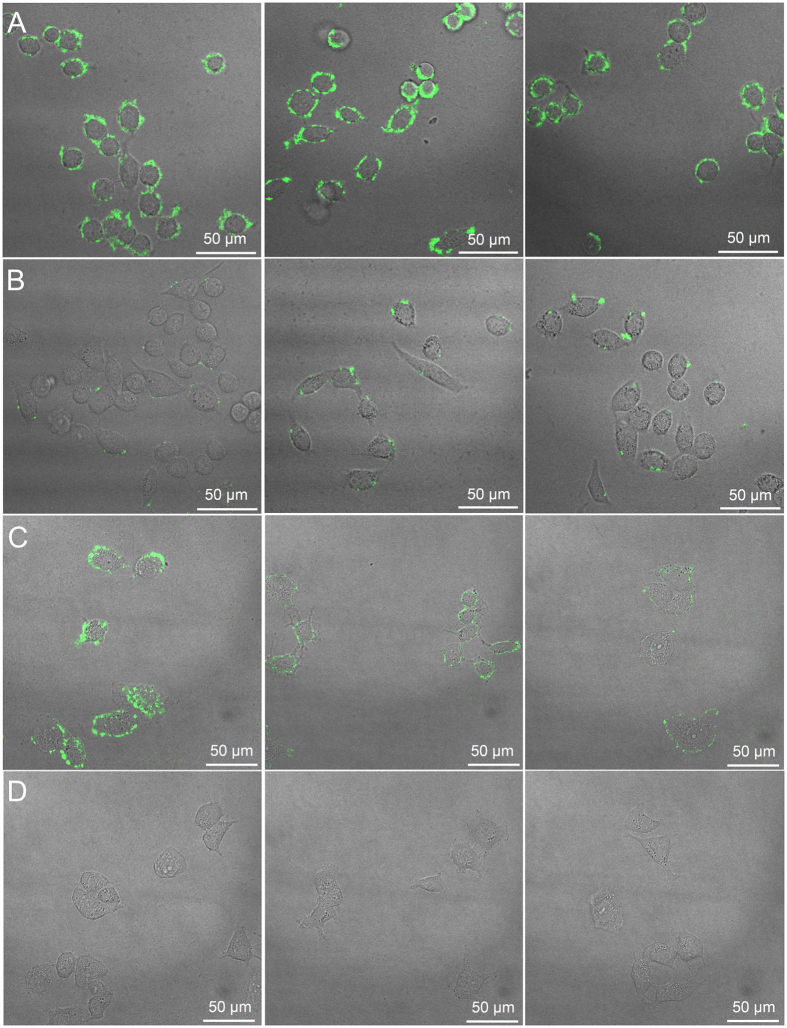
Confocal fluorescence imaging of HepG-2 cells (**A**), L-02 cells (**B**), MCF-7 cells (**C**) and MCF-10A cells (**D**) after staining with different monosaccharide-imprinted NPs. Columns from left to right: SA-, Fuc- and Man-imprinted NPs. The concentration of the NPs was 200 μg/mL.

**Figure 7:**
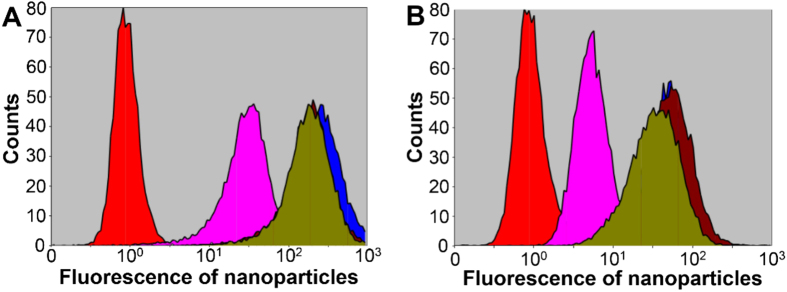
FCM characterization of HepG2 (**A**) and L02 (**B**) after staining with SA-imprinted NPs (200 μg/mL) in the presence of different monosaccharides (200 μg/mL). Red: without staining (controls); pink: SA-imprinted NPs added with SA; blue: SA-imprinted NPs; brown: SA-imprinted NPs added with Man; dark yellow: SA-imprinted NPs added with Fuc.

The synthesis route of monosaccharide-imprinted FITC-doped silica NPs is shown in Fig. 2, which included two procedures: 1) the preparation of FITC-doped SiO_2_ NPs, and 2) the preparation of monosaccharide-imprinted FITC-doped SiO_2_ NPs.

The preparation of FITC-doped SiO_2_ NPs included two steps: 1) the synthesis of FITC-derivatized APTES, and 2) the polycondensation of FITC-derivatized APTES with TEOS. FITC-derivatized APTES was prepared according to a literature method^47^,^48^ with slight modifications. 10 μL APTES was first dissolved into 2 mL ethanol and then 2 mg FITC was added. After reaction for 10 hours, FITC-derivatized APTES formed in the solution. For the polycondensation of FITC-derivatized APTES with TEOS, the FITC-derivatized APTES solution was mixed with 0.48 mL TEOS and 6.4 mL ethanol and the resulting solution was used as precursor. Then, 64 mL ethanol was mixed with 3.88 mL water and 2.88 mL ammonium water. The mixture was slowly heated to 55 °C with vigorous stirring and then rapidly added with the precursor solution. After airtight reaction for 3 hours, FITC-doped SiO_2_ NPs were formed, which were collected via centrifuging. The obtained nanoparticles were washed with ethanol and water twice each. Finally, the prepared FITC-doped SiO_2_ NPs was dispersed into water and stored at room temperature.

The preparation of monosaccharide-imprinted FITC-doped SiO_2_ NPs included four steps: 1) boronic acid functionalization, 2) template immobilization, 3) oriented imprinting, and 4) template removal. To prepare boronic acid-functionalized FITC-doped SiO_2_ NPs, 5 mg/mL FPBA and 1 mg/mL sodium cyanoborohydride were added into a methanol solution containing 10 mg/mL FITC-doped SiO_2_ NPs. After reaction for 24 hours, the resulting boronic acid-functionalized FITC-doped SiO_2_ NPs were collected via centrifuging, and then washed with methanol and water for three times each. The obtained NPs was re-dispersed into water and stored at room temperature. To immobilize the template onto boronic acid-functionalized FITC-doped SiO_2_ NPs, 400 mg monosaccharide template was added into 40 mL phosphate buffer (0.1 M, pH 7.4) containing 1 mg/mL boronic acid-functionalized NPs and the pH was adjusted to 7.4. After incubation for 30 minutes, monosaccharide-bound SiO_2_ NPs were collected via centrifuging and then washed with 0.1 M phosphate buffer (pH 7.4) three times. Monosaccharide-imprinted FITC-doped SiO_2_ NPs were prepared according to the boronate affinity oriented surface imprinting approach reported previously^32^,^39^,^40^,^41^ with major modifications. The monosaccharide-bound FITC-doped SiO_2_ NPs were re-dispersed into 40 mL ethanol, added with 0.7 mL ammonium water and 10 mL prepolymer solution that was consisted of 22.4 μL TEOS and 10 mL ethanol. After reaction for an appropriate duration, the reacting mixture was centrifuged and the precipitates were collected. Finally, to remove the template from the imprinted nanoparticles, the collected precipitates were washed with 0.1 M HAc for 3 hours, followed with 0.1 M phosphate buffer for 30 min and centrifugation again. The obtained monosaccharide-imprinted FITC-doped SiO_2_ NPs were collected and stored in 0.1 M phosphate buffer (pH 7.4).

To prepare non-imprinted SiO_2_ NPs for comparison, the processing procedure was the same except that no template was immobilized onto boronic acid-functionalized SiO_2_ NPs.

### Optimization of the Imprinting Time

The imprinted procedure was the same as described above except that the reaction time was changed. During the reaction, an aliquot of 6 mL was taken out from the reacting mixture every five minutes (six aliquots in total), centrifuged, washed with 0.1 M HAc for 3 hours, and then was centrifuged again. Finally, the monosaccharide-imprinted SiO_2_ NPs were collected and dispersed in 6 mL 0.1 M phosphate buffer (pH 7.4) for further evaluation. Non-imprinted SiO_2_ NPs were also prepared as controls using the same processing procedure except that no template was used.

The monosaccharide-imprinted and non-imprinted SiO_2_ NPs prepared above were evaluated in terms of imprinting factor through the boronate affinity sandwich assay method^20^ using template molecule as a bridge molecule. The above monosaccharide-imprinted and non-imprinted NPs solutions prepared at each imprinting time were added with template molecule (final concentration, 1 mg/mL) and the pH was adjusted to 7.4. After incubation for 30 min, the solutions were centrifuged and the precipitation was rinsed with 0.1 M phosphate buffer (pH 7.4) three times and then dissolved in 6 mL 0.1 M phosphate buffer (pH 7.4). After that, boronic acid-functionalized filter paper pieces were added into each solution and incubated for 30 min. Boronic acid functionalized filter paper pieces incubated with 0.1 M phosphate buffer (pH 7.4) with equal time were used as blanks. After the completion of incubation, all the filter paper pieces were washed with 0.1 M phosphate buffer (pH 7.4) three times. Finally, fluorescence of the resulting filter paper pieces was measured on the microplate reader (each situation tested at least with four pieces of filter paper). IF values were calculated by dividing the fluorescence intensity (blank has been subtracted) of SA-imprinted NPs by that of non-imprinted NPs. Monosaccharide-imprinted NPs prepared at the optimal imprinting time were used for further experiments.

### Adsorption Isotherm and Binding Constant Measurement

A series of SA solutions with known concentrations (0.00625, 0.0125, 0.025, 0.05, 0.1, 0.2, 0.3 and 0.4 mg/mL) were prepared with 0.1 M phosphate buffer (pH 7.4) and their absorbance at 200 nm was measured. 25 mg SA-imprinted and non-imprinted NPs were incubated with 0.5 mL of these SA solutions, respectively. After incubation for 30 min, all the solutions were centrifuged and the absorbance of the supernatants at 200 nm was measured. The SA amounts captured by the imprinted and non-imprinted NPs, which were represented by difference between the absorbance for initial SA solutions and the corresponding supernatants, were plotted against the concentration of the SA solutions. The Hill equation as shown below was used to fit the data for estimation of the binding constant (*K*_d_) of the SA-imprinted NPs toward SA.


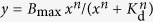


where *B*_max_ is the maximum specific binding, *n* is the Hill slope. Due to the poor UV absorbance of Fuc and Man, however, the measurement of the *K*_d_ values for Fuc- and Man-imprinted NPs was not carried out.

### Selectivity Test

The selectivity was investigated through the boronate affinity sandwich assay^20^ using SA, glucose, fucose, ribose and mannose as bridge molecules, respectively. A 6 × 4 array of a 96-well microplate was used for the experiments. Wells of column from left to right were added with equivalent volumes of 0.1 M phosphate buffer (pH 7.4) without or with 2 mg/mL ribose, fucose, glucose, mannose and SA, respectively. Each well was added with a piece of boronic acid-functionalized filter paper (diameter, 5 mm) and incubated for 30 minutes. After incubation, the filter paper pieces were washed with 0.1 M phosphate buffer (pH 7.4) three times and then added with equivalent volume of 0.1 M phosphate buffer (pH 7.4) containing 1 mg/mL monosaccharide-imprinted SiO_2_ NPs and incubated for 30 minutes. After that, the filter paper pieces were washed with 0.1 M phosphate buffer (pH 7.4) three times. Finally, fluorescence of the filter paper in each well was read on the microplate reader and the fluorescence intensity was blank-subtracted and averaged over each column.

### Cell Imaging

The cell culture medium was removed and the cells remained on the cell culture dishes were washed with 1× PBS for two times. Then the cells were incubated with cell-staining reagents (fluorescent NPs or fluorescein-labeled lectins) dissolved in 1× PBS for certain time. The PBS buffer and free reagents were removed and the remaining cells were rinsed with 1× PBS for three times and supplemented with 1 mL 1× PBS. The obtained cells were imaged under the confocal laser scanning microscope. When boronic acid-functionalized NPs and monosaccharide-imprinted NPs were used for cell staining, their concentration was 200 μg/mL each and the incubation time was 30 min. When fluorescent lectins, including fluorescein-labeled *Sambucus nigra* lectin, fluorescein-labeled *Ulex europaeus* agglutinin I and fluorescein-labeled *Lens culinaris* agglutinin, their concentration was 20 μM and the incubation time was 60 min.

To investigate the influence of the presence of monosacchrides on cell imaging, the cells were respectively stained with boronic acid-functionalized and SA-imprinted NPs in the presence of SA, fucose and mannose (200 μg/mL each) dissolved in 1× PBS for 30 min. Then solution was removed and the remaining cells were rinsed with 1× PBS for three times and supplemented with 1 mL 1× PBS. The obtained cells were imaged under the confocal laser scanning microscope.

### Glycosidase Treatment of Cells

The cell culture medium was removed and the cells remained on the cell culture dishes were washed with 1 × PBS for two times. Then the obtained cells were incubated in culture medium containing 1 unit/mL sialidase, fucosidase or mannosidase for 4 h. After that, the culture medium was removed and the resulting cells were washed with 1 × PBS for three times. Finally, the sialidase-, fucosidase- and mannosidase-treated cells were respectively stained with SA-, Fuc- and Man-imprinted NPs and followed by fluorescence imaging as the procedure described above.

### Flow Cytometry

For flow cytometry assay, the above prepared cells were digested with parenzyme cell digestion solution (containing 0.25% tryptase and 0.02% EDTA) for 2–3 min. The obtained cells were centrifuged at 1,000 rpm for 3 min. After removing the supernatant, the cells were washed with 1× PBS for two times and filtrated with 200 mesh sieves. The obtained cell suspensions were injected into cytoanalyzer and the count of cells was set to 20,000.

## Additional Information

**How to cite this article**: Wang, S. *et al.* Targeting and Imaging of Cancer Cells via Monosaccharide-Imprinted Fluorescent Nanoparticles. *Sci. Rep.* **6**, 22757; doi: 10.1038/srep22757 (2016).

## Supplementary Material

Supplementary Information

## Figures and Tables

**Figure 1:**
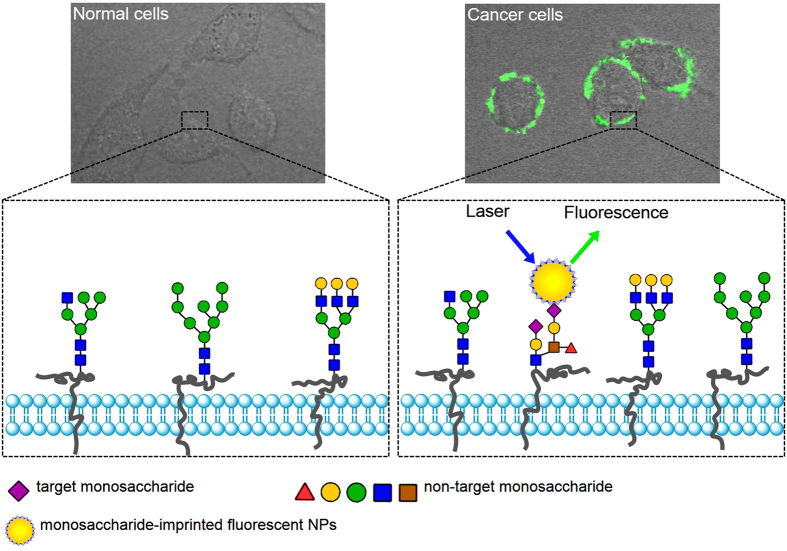
Schematic of the targeting and imaging of cancer cells with monosaccharide-imprinted NPs.
