# Supplementary material for: Targeting and Imaging of Cancer Cells via Monosaccharide-Imprinted Fluorescent Nanoparticles
Source: Sci Rep. 2016 Mar 7;6:22757. doi: 10.1038/srep22757 (PMC4780104; doi:10.1038/srep22757)
Supplement: Supplementary Information [file srep22757-s1.pdf]

Supplementary Information for

Targeting and Imaging of Cancer Cells via Monosaccharide-Imprinted Fluorescent Nanoparticles

Shuangshou Wang[1,2], Danyang Yin[1,2], Wenjing Wang[1,2], Xiaojing Shen[1,2], Jun-Jie Zhu[1,2], Hong-Yuan Chen[1,2] & Zhen Liu[1,2]

[1] State Key Laboratory of Analytical Chemistry for Life Science, School of Chemistry and Chemical Engineering, Nanjing University, Nanjing 210023, China. [2] Collaborative Innovation Center of Chemistry for Life Sciences, School of Chemistry and Chemical Engineering, Nanjing University, Nanjing 210023, China.

Correspondence and requests for materials should be addressed to Z. L. (Email: zhenliu@nju.edu.cn)

Supplementary Experimental Details

Materials and Chemicals. 3-Aminopropyltriethoxysilane (APTES, 98%), fluorescein isothiocyanate isomer (FITC, ≥90%), dimethyl sulfoxide (DMSO, 99%), sialidase, α-L-fucosidase, and α-mannosidase were purchased from Sigma Aldrich (St. Louis, MO, USA). L-fucose (Fuc, 99%), alizarin red S (ARS, HPLC grade) and *N*-acetylneuraminic acid (SA, 98%) were purchased from Aladdin Industrial (Shanghai, China). 4-Formylphenylboronic acid (FPBA, 97%), sodium cyanoborohydride (95%) and D-glucose (99%) were purchased from J&K Chemical (Shanghai, China). D-mannose (Man, 99%) was purchased from Alfar Aesar (Tianjin, China). *N*-aminoethyl-γ-aminopropyltriethoxy-silane, D-ribose (99%), and ammonia water (28% w) were purchased from Sinopharm Chemical Reagent (Shanghai, China). Fluorescein-labeled lectins, including *Lens culinaris* agglutinin, *Ulex europaeus* agglutinin I and *Sambucus nigra* lectin, were purchased from Vector Laboratories (Burlingame, USA). Tetraethyl orthosilicate (TEOS, 99%) was purchased from Heowns Biochemical Technology (Tianjin, China). Filter paper was purchased from Whatman (Hangzhou, China). Hepatoma carcinoma cell (HepG-2), normal hepatocyte cell (L-02), mammary cancer cell (MCF-7), normal mammary epithelial cell (MCF-10A), phosphate buffer solution for cell culture (1×PBS), parenzyme cell digestion solution (containing 0.25% trypase and 0.02% EDTA) and Dulbecco Modified Eagle Medium (DMEM, containing 4.5 mg/mL glucose, 80 U/mL penicillin and 0.08 mg/mL streptomycin) were purchased from Keygen Biotech

(Nanjing, China). Fetal bovine serum (FBS) was purchased from Gibco (Life Technologies, Australia). All other chemical reagents were of analytical grade unless otherwise noted. Water used in all experiments was purified by a Milli-Q Advantage A10 ultrapure water purification system (Millipore, Milford, MA, USA). Glass bottom cell culture dishes (Φ 20 mm) obtained from NEST Biotechnology (Wuxi, China) were used for cell culture and imaging.

Apparatus. Transmission electron microscopy (TEM) was performed on a JEOL JEM-1011 TEM instrument (Tokyo, Japan). Characterization of particle size was carried out on a BI-200SM dynamic light scattering (DLS) machine (Brookhaven Instrument, Holtsville, USA) instrument. Fluorescence properties of the prepared nanoparticles as well as FITC were characterized on a RF-5301PC fluorescence spectrometer (Shimadzu China, Shanghai, China). Evaluation of boronic acid functionalization and molecular imprinting was performed on a synergy Mx microplate reader from BioTek (Winooski, VT, USA). UV-vis absorption characterization was carried out on a Nanodrop-2000C instrument (Thermo Fisher Scientific, Shanghai, China). Cell imaging was performed on a Leica TCS SP5 confocal laser scanning microscope (Leica Microsystems CMS GmbH, Mannheim, Germany). Flow cytometry (FCM) was performed on a Cytomics FC500 cytoanalyzer (Beckman Coulter, Indianapolis, IN, USA).

Leakage Test of FITC-doped SiO_2 NPs. 10 mg FITC-doped SiO_2 NPs was dispersed into 20 mL water and ultrasonicated continuously. A 3-mL aliquot was taken out every

one hour from the solution (five aliquots in total) and centrifuged, then the supernatant was collected and the precipitation was re-dispersed in 3 mL water. Fluorescence of the supernatant and re-dispersed nanoparticles was measured separately.

Boronate Affinity Sandwich Assay. Since the SA-imprinted and non-imprinted nanoparticles were both fluorescent while the direct photometric detection of monosaccharides was difficult, a boronate affinity sandwich assay[1] was introduced to evaluate the boronic acid functionalization of FITC-doped SiO_2 NPs and relevant properties of the imprinted nanoparticles, such as imprinting factor (IF), selectivity and binding dynamics. Briefly, a piece of boronic acid-functionalized filter paper was used as a substrate, a monosaccharide containing dual cis-diol moieties (such as glucose and sialic acid) was used as bridge molecule to bind with the substrate and SiO_2 NPs. After excessive nanoparticles removed through washing with 0.1 M phosphate buffer (pH 7.4), the fluorescence intensity of the formed filter paper-monosaccharide-nanoparticle sandwiches was detected with excitation at 495 nm and emission at 520 nm for the FITC-doped nanoparticles.

To prepare boronic acid-functionalized filter papers, filter papers were immersed into 50-fold diluted piranha solution for 10 hours, rinsed with ethanol three times, and then further immersed into an ethanol solution containing 5% (v/v) *N*-aminoethyl-γ-aminopropyltriethoxysilane for reaction at 60 °C for 10 hours. The filter papers were washed with ethanol three times, and then further immersed into an ethanol solution containing FPBA and sodium cyanoborohydride (5 mg/mL each) and reacted at 60 °C for

another 10 hours. Finally, the filter papers were washed with ethanol and water three times each, air dried and sealed at room temperature for further use.

To characterize the boronic acid-functionalization of filter papers, boronic acid-functionalized and non-functionalized filter papers were cut into small pieces (diameter, 5 mm) by punching. A 4×4 array of a 96-well microplate was used for the experiments. Equivalent volumes of phosphate buffer (0.1 M, pH 7.4) and ARS solution (0.1 mg/mL, dissolved in 0.1 M phosphate buffer, pH 7.4) were added to column A, C (both used as blank) and column B, D, respectively. Column A and B were added with non-functionalized filter paper pieces while C and D were added with boronic acid-functionalized filter paper pieces (1 piece per well). After incubation at room temperature for 30 minutes, all the filter paper pieces were rinsed with 0.1 M phosphate buffer (pH 7.4) and the fluorescence intensity of the resulting filter paper pieces was measured on microplate reader under excitation wavelength of 530 nm and emission wavelength of 630 nm. Blank fluorescence signals were subtracted from the fluorescence intensities and the obtained intensities for each column were averaged.

Evaluation of Boronic Acid-functionalization of SiO_2 NPs. Small pieces of boronic acid-functionalized filter papers were incubated with glucose solution (1 mg/mL, dissolved in 0.1 M phosphate buffer, pH 7.4) for 30 min. The glucose-bound filter paper pieces were washed with phosphate buffer (0.1 M, pH 7.4) three times. Then, the filter paper pieces were added into three different solutions and incubated for 30 min: 1)

phosphate buffer (0.1 M, pH 7.4) containing 1 mg/mL boronic acid-functionalized SiO_2 NPs; 2) phosphate buffer (0.1 M, pH 7.4) containing 1 mg/mL non-functionalized SiO_2 NPs; and 3) phosphate buffer (0.1 M, pH 7.4) without the presence of any NPs (used as blank). All the filter paper pieces were washed with phosphate buffer (0.1 M, pH 7.4) three times. Finally, fluorescence of the filter papers was measured on the microplate reader (each situation was tested at least with three pieces of filter paper). Blank fluorescence signals were subtracted from the fluorescence intensities and the obtained intensities for boronic acid-functionalized and non-functionalized SiO_2 NPs were averaged.

Imprinting Efficiency. The absorbance at 200 nm of 0.4 mg/mL SA solution containing 0.1 M phosphate buffer (pH 7.4) was measured. 25 mg SA-imprinted and boronic acid-functionalized NPs were respectively incubated with 0.5 mL of above SA solution for 30 min. After centrifuging, the absorbance at 200 nm of the resulting supernatants was measured. The difference in the absorbance of the SA solution subtracted by the absorbance of the corresponding supernatant was used to represent the saturated adsorbed SA amount by SA-imprinted and boronic acid-functionalized NPs, respectively. The IE value was calculated by dividing the saturated adsorbed SA amount of SA-imprinted NPs by that of the boronic acid-functionalized NPs. The measurement was repeated with three times. Due to the poor UV absorbance of Fuc and Man, however, the measurement of the imprinting efficiency for Fuc- and Man-imprinted NPs was not carried out.

Binding Dynamics Test. The binding dynamics was investigated through the boronate affinity sandwich assay[1] using SA as a bridge molecule. A 7×4 array of a 96-well microplate was used for this experiment. 40 mg SA was dissolved into 40 mL 0.1 M phosphate buffer containing 1 mg/mL SA-imprinted SiO_2 NPs and the pH was adjusted to 7.4. An aliquot of 5 mL was taken out of the solution at incubation time of 10, 15, 20, 25, 30, 40, and 50 min, centrifuged and washed with 0.1 M phosphate buffer (pH 7.4) three times each. The resulting precipitation was re-dispersed in 5 mL 0.1 M phosphate buffer (pH 7.4). Column from left to right of the micro-well array was respectively added with the re-dispersed solutions obtained at different incubation times, and then added with boronic acid-functionalized filter paper piece (one piece per well). After incubation for 30 min, the resulting filter paper pieces were rinsed with 0.1 M phosphate buffer (pH 7.4) three times. Finally, fluorescence of different filter papers was measured on the microplate reader and averaged over each column.

Cell Culture. Cells under investigation, including HepG-2, L-02, MCF-7 and MCF-10A cells, were cultured in DMEM medium with 10% fetal bovine serum for 2 to 3 days (37 °C, 5% CO_2).

Reference

1. Ye, J., Chen, Y. & Liu, Z. A boronate affinity sandwich assay: an appealing alternative to immunoassays for the determination of glycoproteins. *Angew. Chem. Int. Ed.* **53**, 10386-10389 (2014).

Supplementary Figures

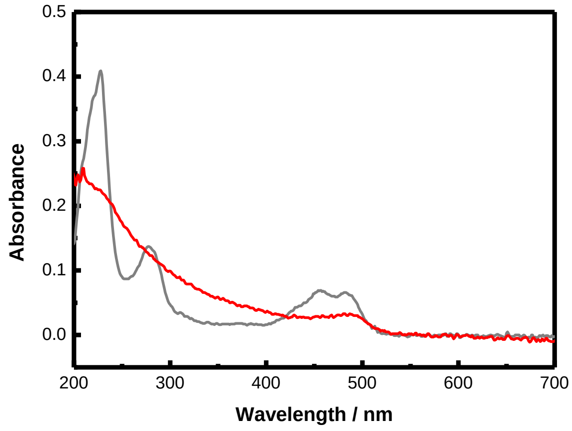

Figure S1. UV-vis absorbance spectra of FITC molecules (gray) and FITC-doped NPs (red). Sample: 0.01 mg/mL FITC or 2.0 mg/mL FITC-doped silica NPs dissolved in water.

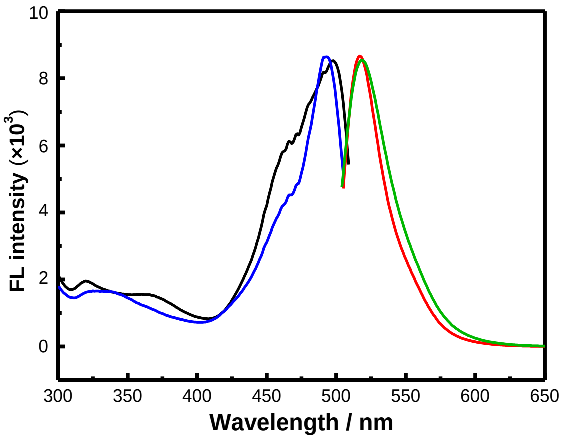

Figure S2. Fluorescence spectrum of FITC-doped NPs and FITC molecules. Black: excitation of FITC-doped NPs; red: emission of FITC-doped NPs; blue: excitation of FITC molecules; green: emission of FITC molecules. Sample: 1 mg/mL FITC-doped SiO_2 NPs or 0.025 mg/mL FITC dissolved in water.

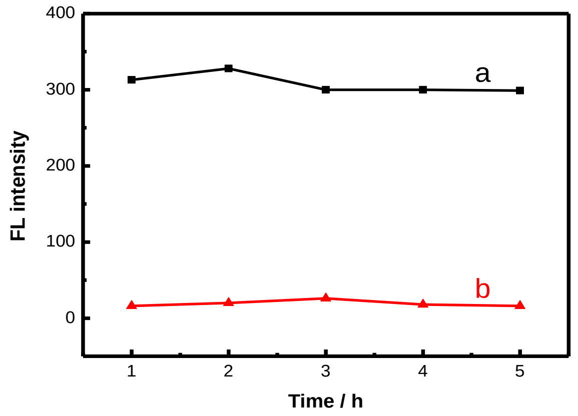

Figure S3. The fluorescence intensity of FITC-doped SiO_2 NPs (a) and supernatant from centrifuged FITC-doped SiO_2 NPs water solution (b) at different times during continuous ultrasonication. Excitation wavelength: 495 nm; emission wavelength, 520 nm.

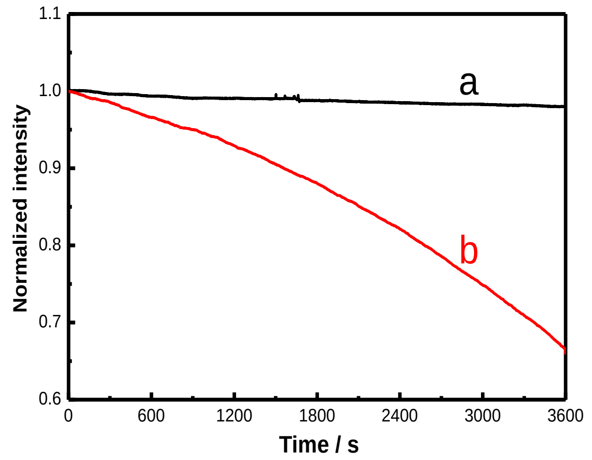

Figure S4. Photostability of FITC-doped silica NPs (a) and FITC molecules (b). Solutions containing 1 mg/mL FITC-doped SiO_2 NPs and 0.01 mg/mL FITC molecules respectively were continuously excited at 495 nm for 1 hour and the fluorescence emitted at 520 nm was recorded.

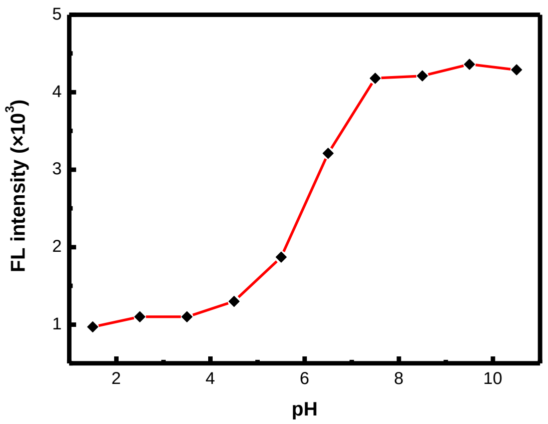

Figure S5. Dependence of the fluorescence intensity of FITC-doped SiO_2 NPs on surrounding pH. Concentration: 1 mg/mL dissolved in 0.1 M phosphate solution at different pH; excitation wavelength: 495 nm; emission wavelength, 520 nm.

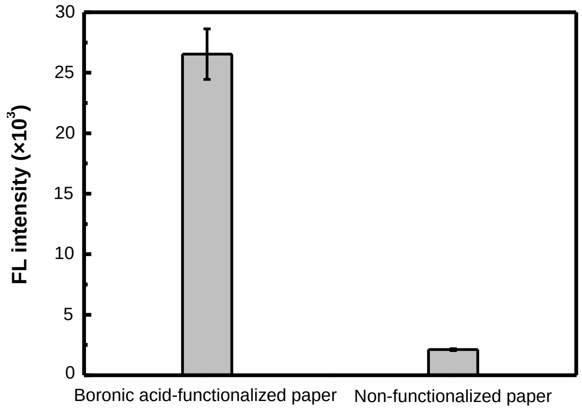

Figure S6. Fluorescence intensity of boronic acid-functionalized filter papers and non-functionalized filter papers after incubating with ARS solution (0.1 mg/mL, dissolved in 0.1 M phosphate buffer, pH 7.4). Excitation wavelength: 530 nm; emission wavelength, 630 nm.

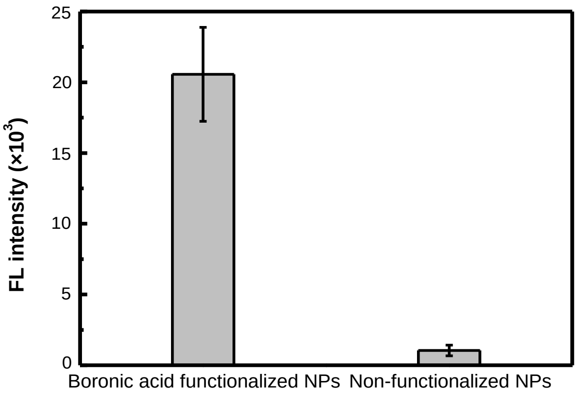

Figure S7. Fluorescence intensity of boronic acid functionalized NPs and non-functionalized NPs detected through the boronate affinity sandwich assay method using glucose as a bridge molecule.

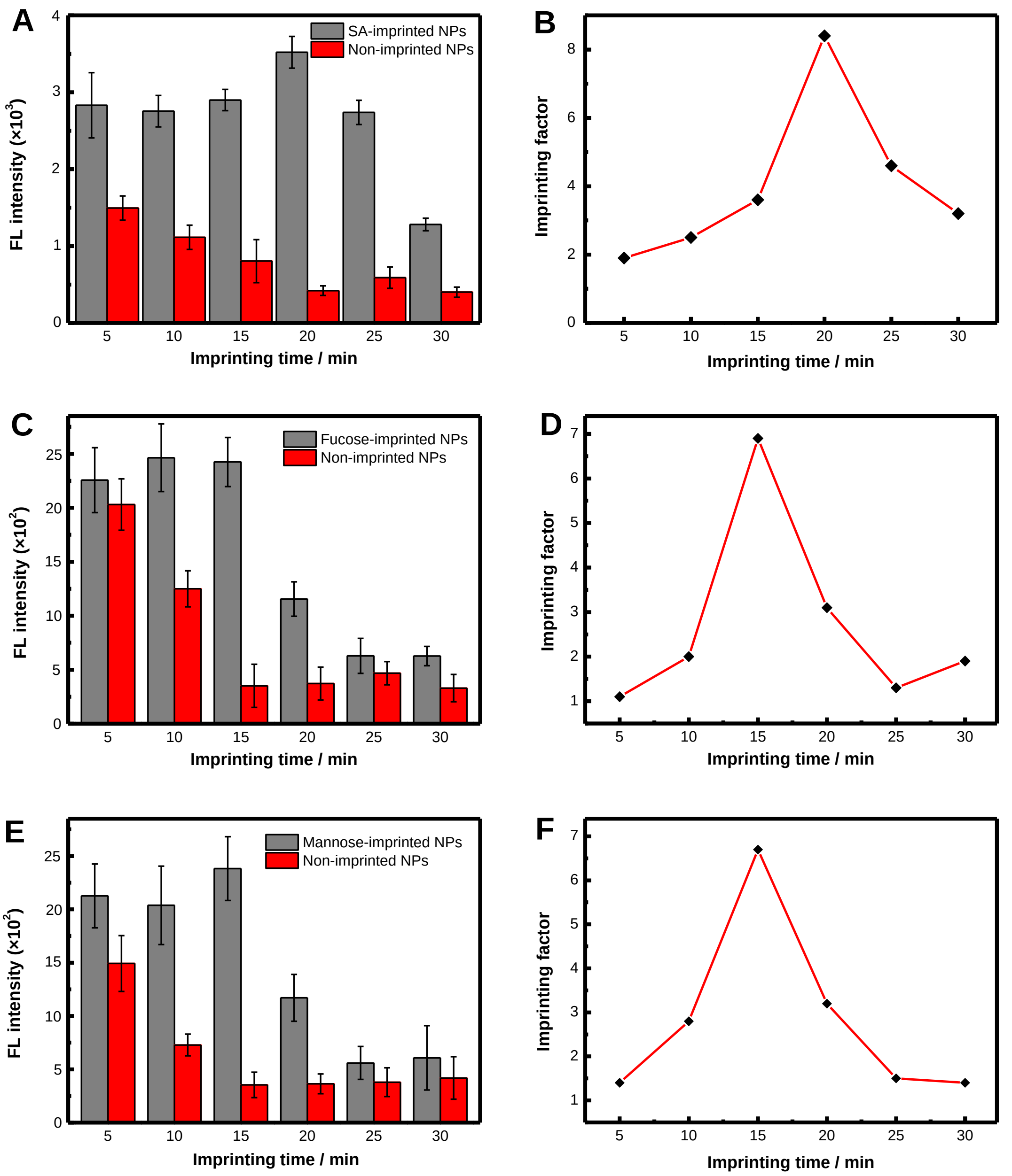

Figure S8. Dependence of the fluorescence intensity of SA- (A), Fuc- (C), and Man-imprinted (E) NPs and corresponding non-imprinted NPs on imprinting time; Dependence of the imprinting factor of SA- (B), Fuc- (D), and Man-imprinted (F) NPs on imprinting time.

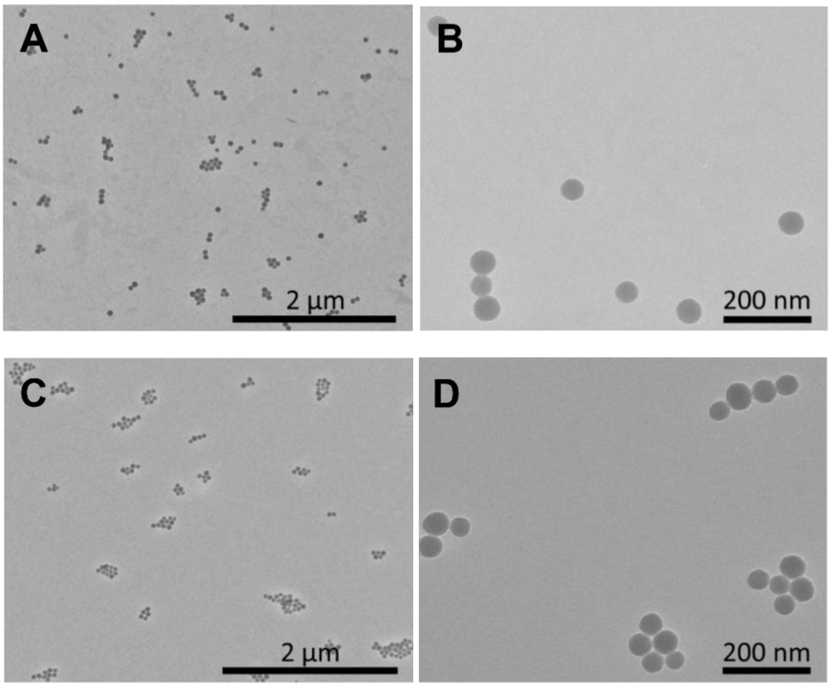

Figure S9. TEM characterization of SA-imprinted (A and B) and non-imprinted NPs (C and D) (both SA-imprinted and non-imprinted NPs were prepared under the optimized imprinting time of 20 min).

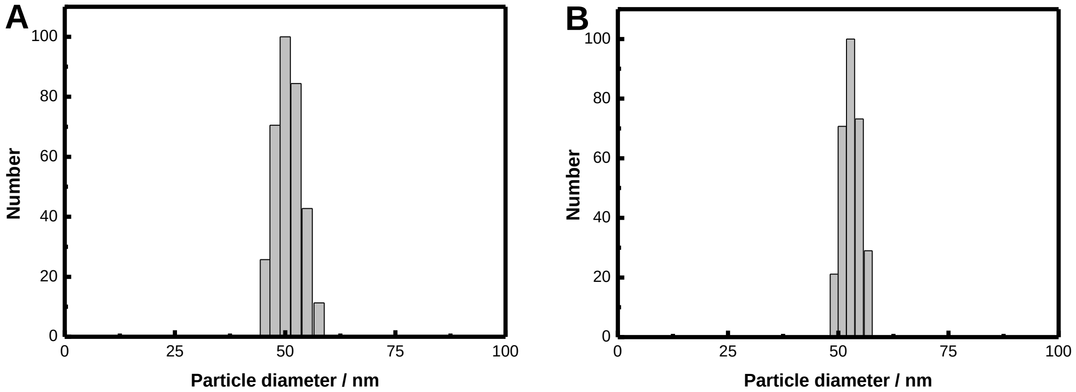

Figure S10. DLS characterization of SA-imprinted (A) and non-imprinted NPs (B) (both SA-imprinted and non-imprinted NPs were prepared under the optimized imprinting time of 20 min).

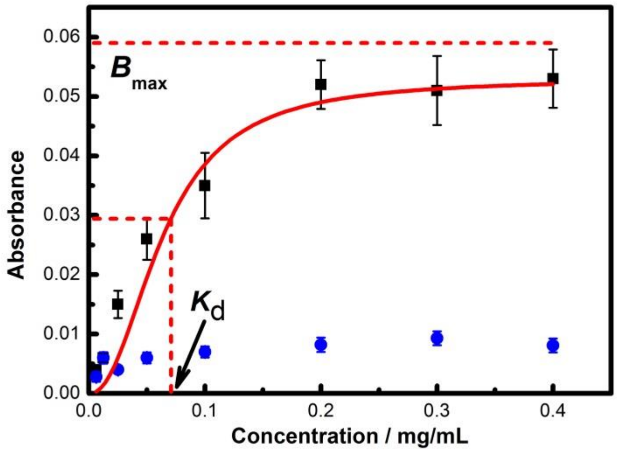

Figure S11. Adsorption isotherms of SA on SA-imprinted (black) and non-imprinted (blue) NPs. Red curve, data fitting by the Hill equation.

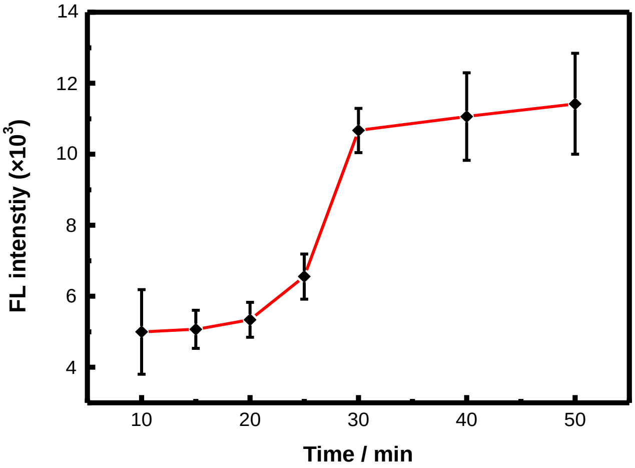

Figure S12. Dependence of the fluorescence intensity of SA-imprinted NPs on the incubation time with SA.

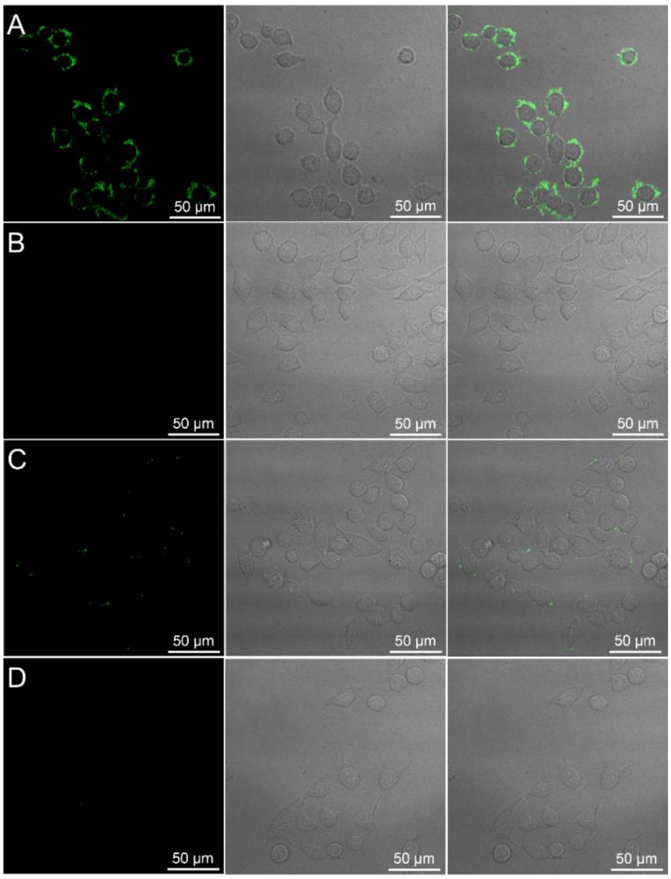

Figure S13. Confocal fluorescence imaging of HepG-2 cells (A, B) and L-02 cells (C, D) after staining with SA-imprinted NPs (A, C) and non-imprinted NPs (B, D). Columns from left to right: dark, bright and overlaid field.

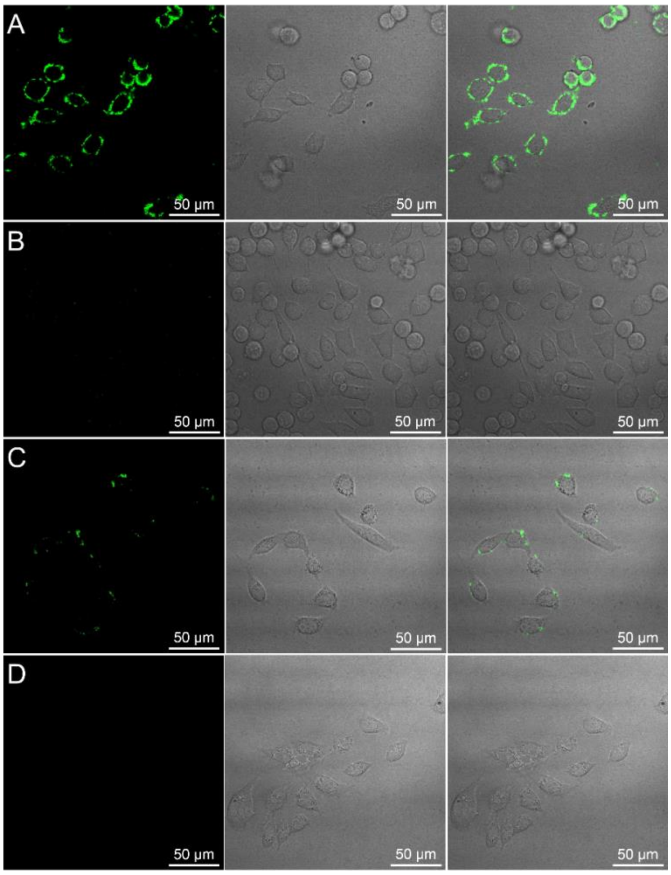

Figure S14. Confocal fluorescence imaging of HepG-2 cells (A, B) and L-02 cells (C, D) after staining with Fuc-imprinted NPs (A, C) and non-imprinted NPs (B, D). Columns from left to right: dark, bright and overlaid field.

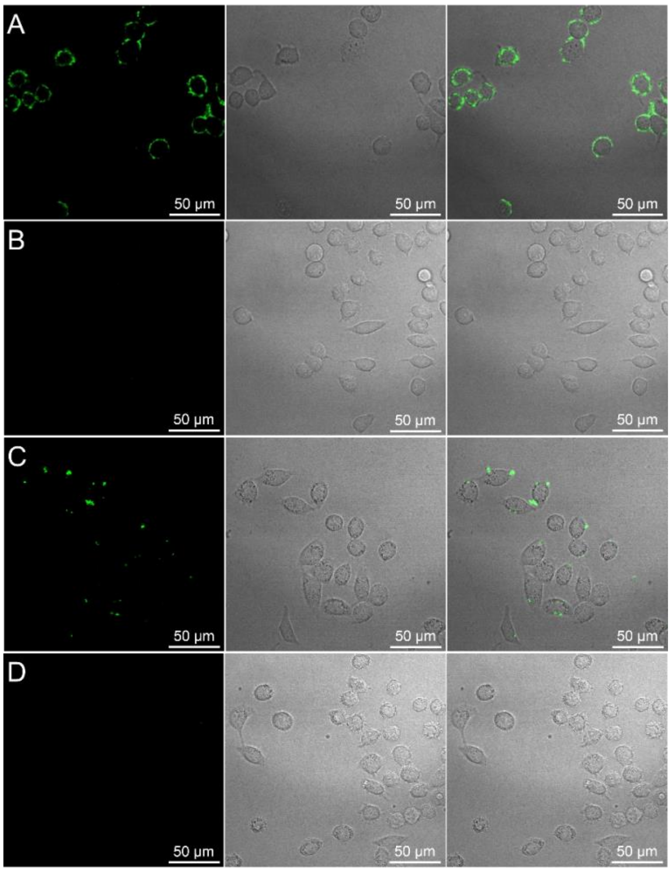

Figure S15. Confocal fluorescence imaging of HepG-2 cells (A, B) and L-02 cells (C, D) after staining with Man-imprinted NPs (A, C) and non-imprinted NPs (B, D). Columns from left to right: dark, bright and overlaid field.

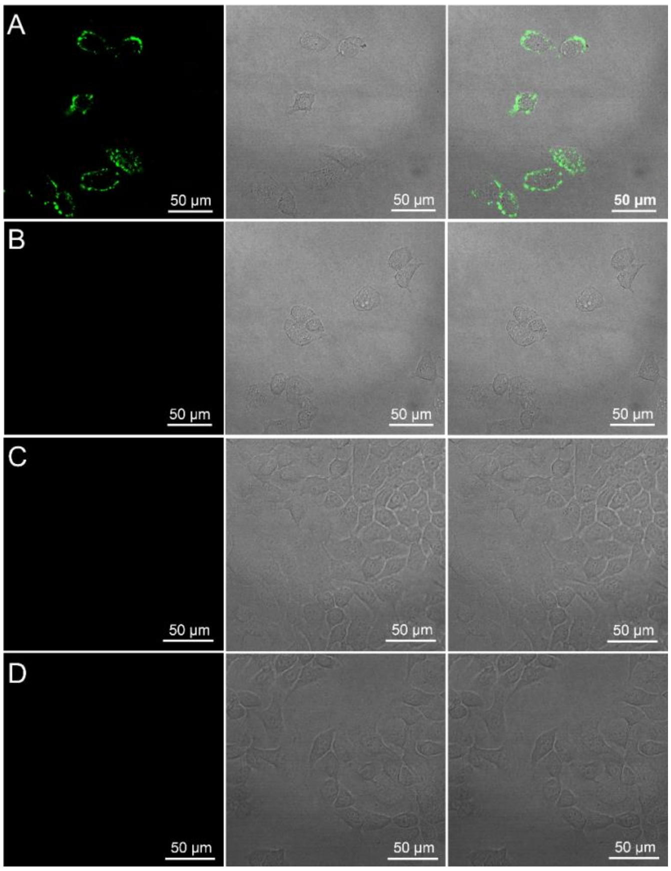

Figure S16. Confocal fluorescence imaging of MCF-7 (A, B) and MCF-10A (C, D) cells after staining with SA-imprinted NPs (A, C) and non-imprinted NPs (B, D). Columns from left to right: dark, bright and overlaid field.

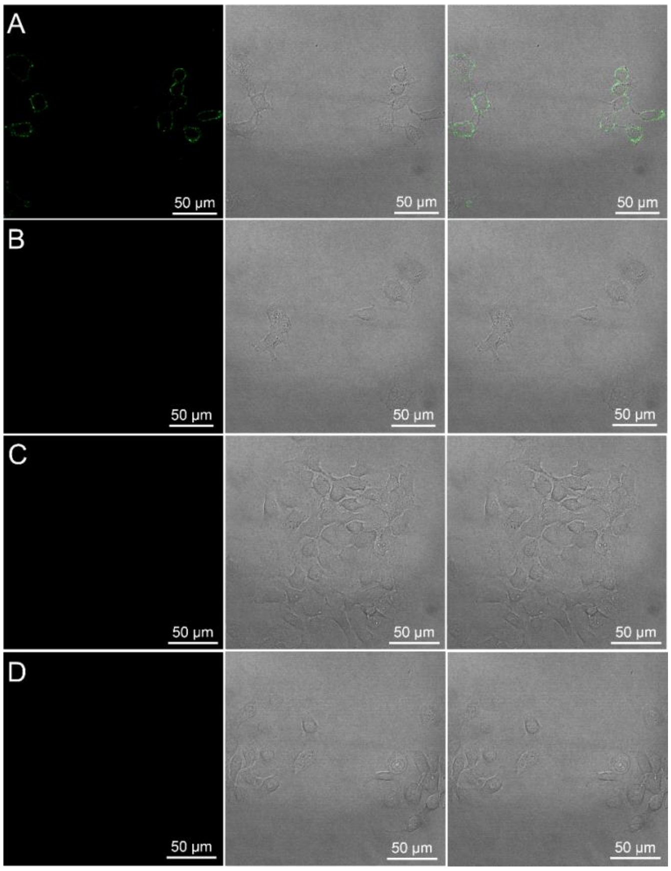

Figure S17. Confocal fluorescence imaging of MCF-7 (A, B) and MCF-10A (C, D) cells after staining with Fuc-imprinted NPs (A, C) and non-imprinted NPs (B, D). Columns from left to right: dark, bright and overlaid field.

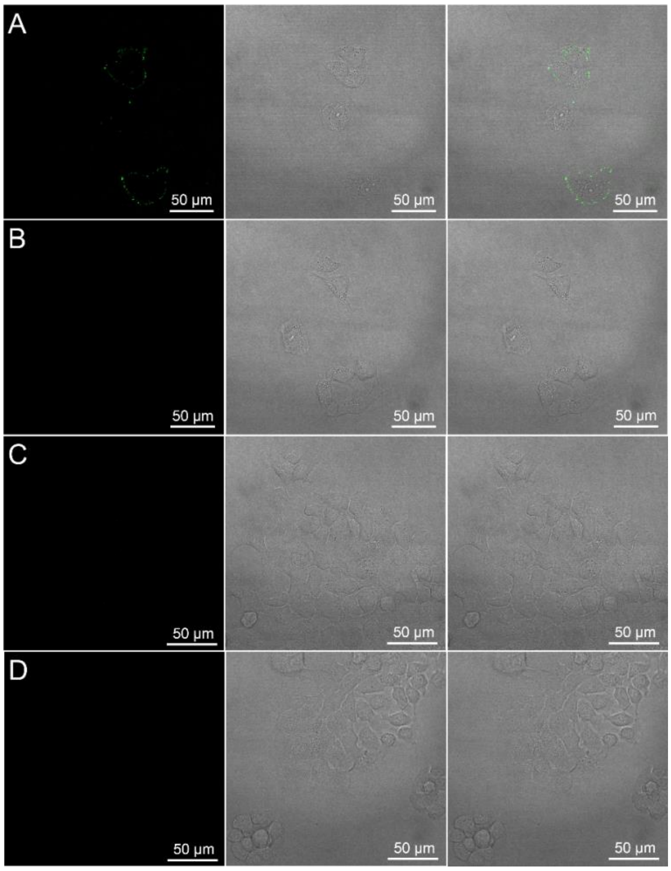

Figure S18. Confocal fluorescence imaging of MCF-7 (A, B) and MCF-10A (C, D) cells after staining with Man-imprinted NPs (A, C) and non-imprinted NPs (B, D). Columns from left to right: dark, bright and overlaid field.

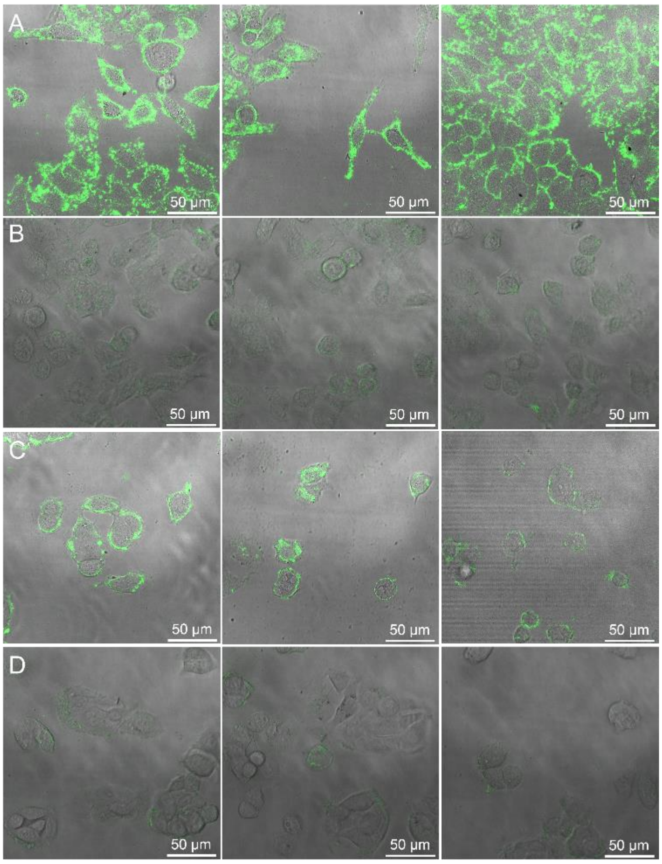

Figure S19. Confocal fluorescence imaging of HepG-2 (A), L-02 (B), MCF-7 (C) and MCF-10A (D) cells after staining with fluorescein-labeled *Sambucus nigra* lectin (left column), fluorescein-labeled *Ulex europaeus* agglutinin I (middle column) and fluorescein-labeled *Lens culinaris* agglutinin (right column), respectively. Concentration of the lectins, 20 μM.

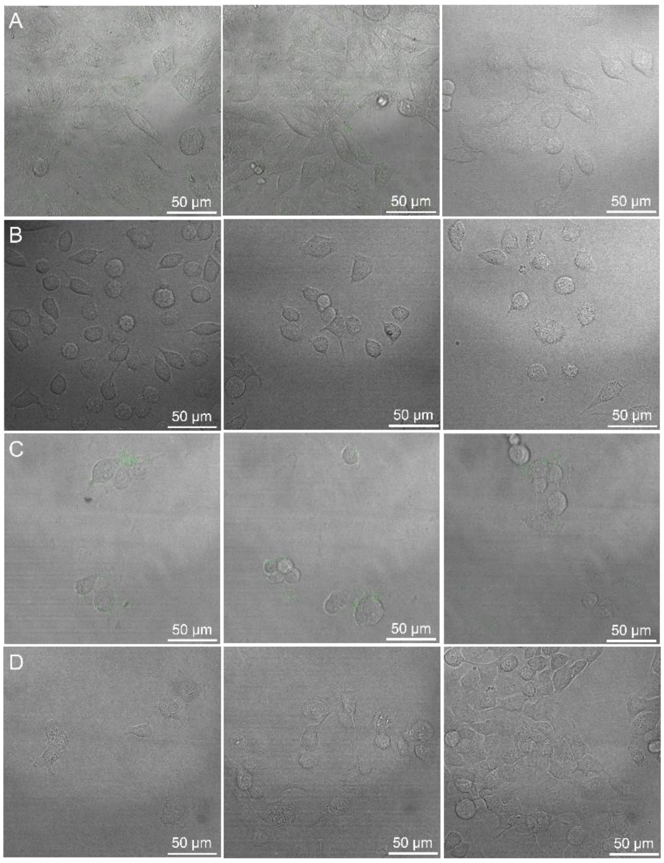

Figure S20. Confocal fluorescence imaging of HepG-2 (A), L-02 (B), MCF-7 (C) and MCF-10A (D) cells after the cells were treated with sialidase (left column), fucosidase (middle column) and mannosidase (right column). Cell staining NPs: SA-imprinted NPs (left column), Fuc-imprinted NPs (middle column) and Man-imprinted NPs (right column). Concentration of the enzymes, 1 unit/mL.

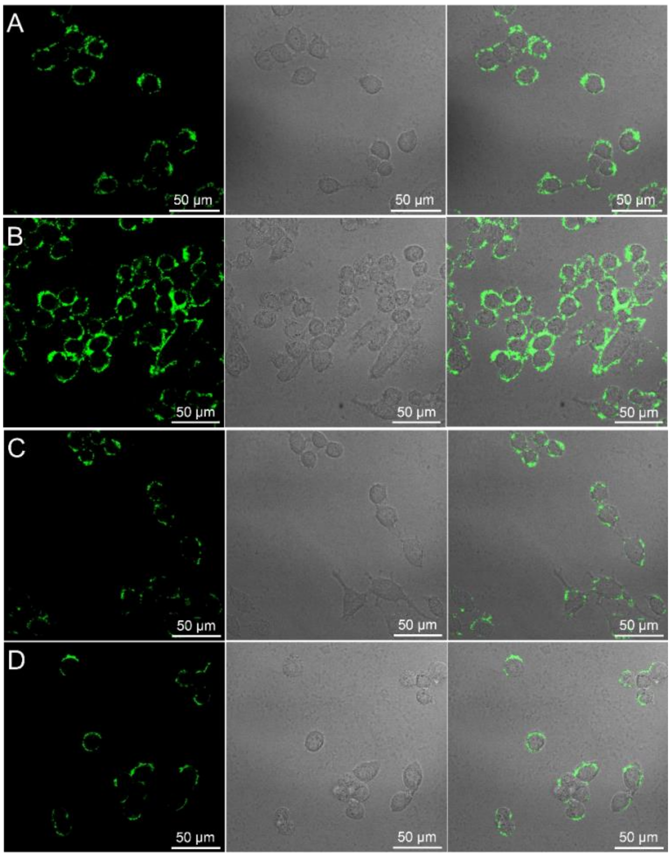

Figure S21. Confocal fluorescence imaging of HepG-2 cells (A, C) and L-02 cells (B, D) after staining with boronic acid-functionalized NPs with different concentrations. Concentration: 200 μg/mL for A and B; 100 μg/mL for C and D. Columns from left to right: dark, bright and overlaid field.

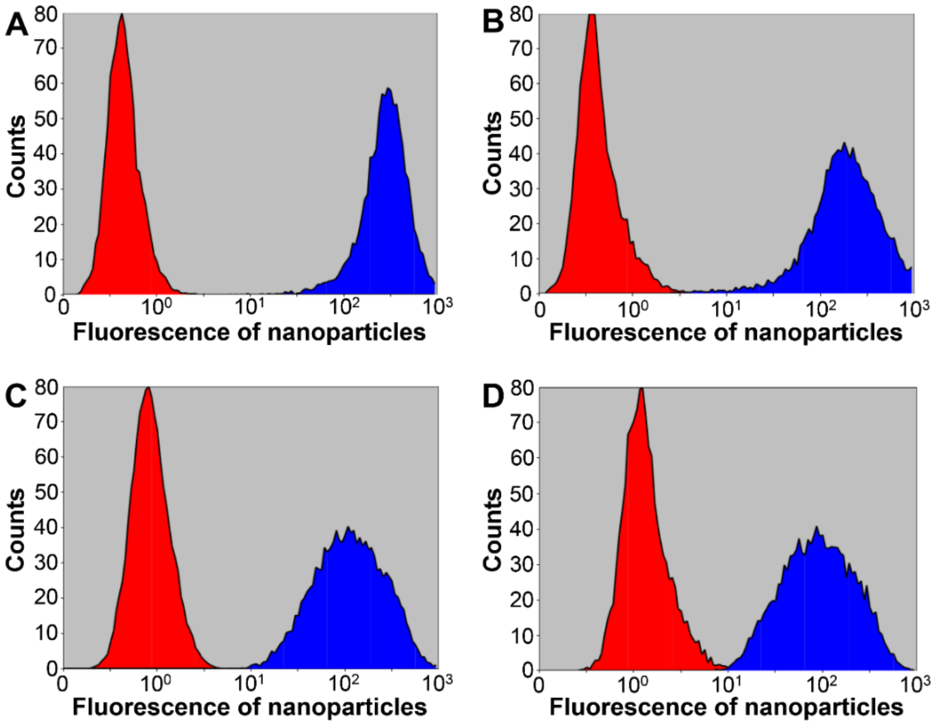

Figure S22. FCM characterization of HepG-2 cells (A), L-02 cells (B) and mixed cells (C and D) after staining with boronic acid-functionalized NPs (200 μg/mL). The ratio of HepG-2 cells to L-02 cells for C and D was 1:1 and 1:5 respectively. Red: without staining (controls); blue: stained with boronic acid-functionalized NPs.

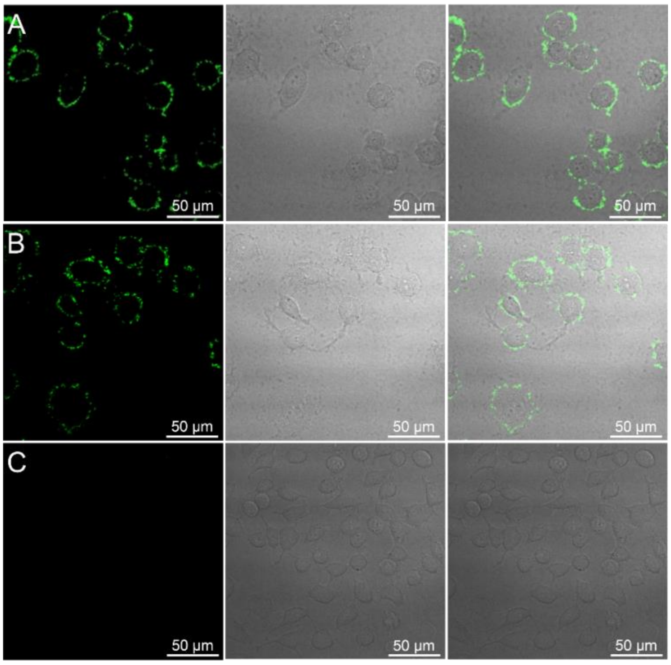

Figure S23. Confocal fluorescence imaging of HepG-2 cell after staining with SA-imprinted NPs (200 μg/mL) in the presence of different monosaccharides (200 μg/mL). A: Fuc; B: Man; C: SA. Columns from left to right: dark, bright and overlaid field.

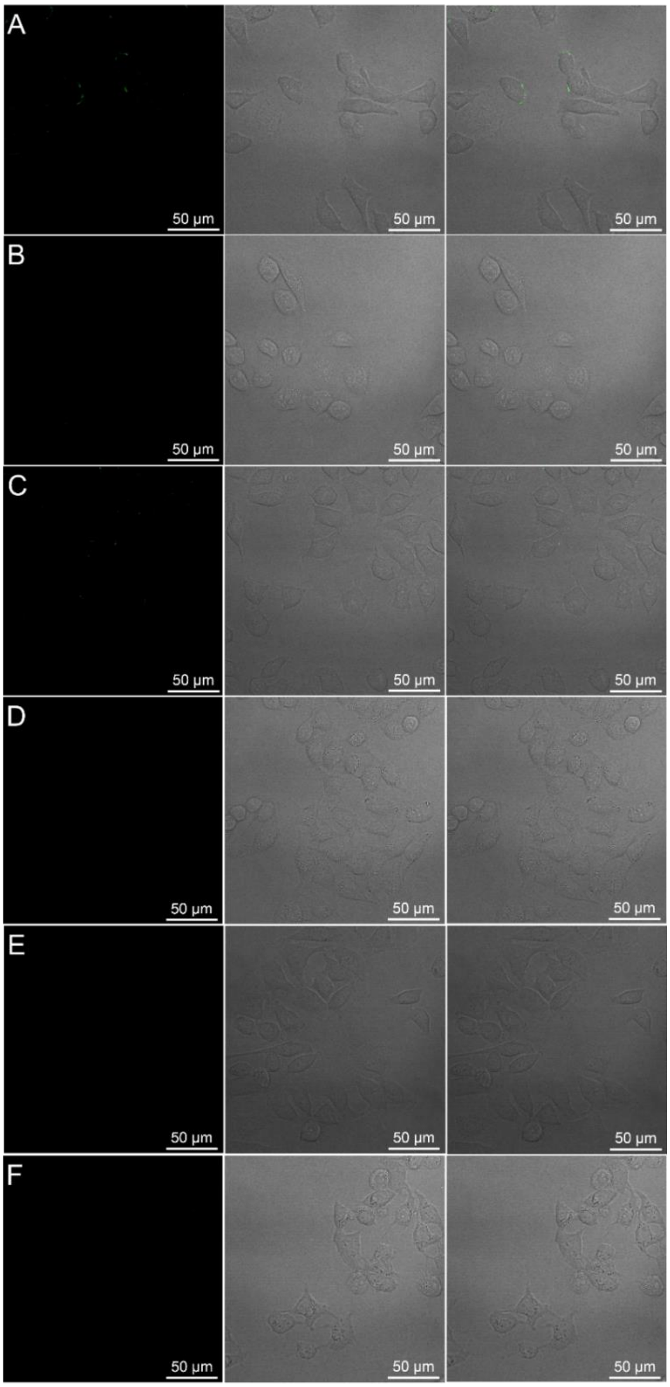

Figure S24. Fluorescence imaging of HepG-2 cells (A, C, E) and L-02 cells (B, D, F) after staining with boronic acid functionalized NPs (200 μg/mL) in the presence of different monosaccharides (200 μg/mL). A and B: SA; C and D: Fuc; E and F: Man. Columns from left to right: dark, bright and overlaid field.
